# Global informetric perspective studies on translational medical research

**DOI:** 10.1186/1472-6947-13-77

**Published:** 2013-07-26

**Authors:** Qiang Yao, Peng-Hui Lyu, Fei-Cheng Ma, Lan Yao, Shi-Jing Zhang

**Affiliations:** 1 School of Medicine and Health Management, Tongji Medical College, Huazhong University of Science and Technology, Wuhan 430030, People’s Republic of China; 2 Center for Studies of Information Resources, Wuhan University, Wuhan 430072, People’s Republic of China

**Keywords:** Translational medical research, Web of science, Informetrics, Social network analysis

## Abstract

**Background:**

Translational medical research literature has increased rapidly in the last few decades and played a more and more important role during the development of medicine science. The main aim of this study is to evaluate the global performance of translational medical research during the past few decades.

**Methods:**

Bibliometric, social network analysis, and visualization technologies were used for analyzing translational medical research performance from the aspects of subject categories, journals, countries, institutes, keywords, and MeSH terms. Meanwhile, the co-author, co-words and cluster analysis methods were also used to trace popular topics in translational medical research related work.

**Results:**

Research output suggested a solid development in translational medical research, in terms of increasing scientific production and research collaboration. We identified the core journals, mainstream subject categories, leading countries, and institutions in translational medical research. There was an uneven distribution of publications at authorial, institutional, and national levels. The most commonly used keywords that appeared in the articles were “translational research”, “translational medicine”, “biomarkers”, “stroke”, “inflammation”, “cancer”, and “breast cancer”.

**Conclusions:**

The subject categories of “Research & Experimental Medicine”, “Medical Laboratory Technology”, and “General & Internal Medicine” play a key role in translational medical research both in production and in its networks. *Translational medical research and CTS, etc.* are core journals of translational research. G7 countries are the leading nations for translational medical research. Some developing countries, such as P.R China, also play an important role in the communication of translational research. The USA and its institutions play a dominant role in the production, collaboration, citations and high quality articles. The research trends in translational medical research involve drug design and development, pathogenesis and treatment of disease, disease model research, evidence-based research, and stem and progenitor cells.

## Background

Translational medical research, originated from the concept of B2B (bench-to-bedside), is a class of medical research aiming to eliminate the barriers between laboratory and clinical research [1]. It converts promising laboratory discoveries into clinical applications and elucidates clinical questions with the use of bench work, aiming to facilitate prediction, prevention, diagnosis, and treatment of diseases [2,3]. Translational medical research is defined as “the application of discoveries generated by laboratory research and preclinical studies to the development of clinical trials and studies in humans and a second area of translational research concerns enhancing the adoption of best practices” in MeSH [4]. In addition, Elias A.Zerhouni, the director of the National Institutes of Health (NIH), put forward the concept of “translational medicine” in the NIH Roadmap in 2003. The core of the concept is to transform the basic research achievements of medical biology into practical theory, technology and methods that will bridge laboratory and clinical practice [2].

The US National Institutes of Health (NIH) has devoted considerable resources to establish translational research centers, hence medical schools and universities have developed many translational research training programs. New medical journals specifically designed to cover translational science are rising at an astonishing pace. Yet clear and coherent definitions of translational medicine are lacking because translational medicine means different things to different people [5]. To molecularly based scientists, it means bridging the gap between basic and clinical sciences, i.e., transforming knowledge derived through basic science investigation into improved diagnosis and treatment of patients in a bench-to-bedside flow of information. To health care delivery scientists, translational medicine means translating knowledge about individuals into populations, to close the gap in the access and delivery of new treatment options [5]. The literature today includes a plethora of attempts in various fields to define the term, and many “T’s” modes in translational medical research such as 2 T, 3 T, and 4 T modes were formed during its development periods. The Institute of Medicine Clinical Research Roundtable first described the current terminology and model of translational research in 2003 as a two-phase process of research progressing from (1) basic science to clinical science, and (2) from clinical science to public health impact. In this framework, they identified “translational blocks” in more steps. The obstacles to research progress represent major challenge areas for obtaining health improvements from the basic sciences in its field. The first roadblock (T1) was described by the roundtable as “the transfer of new understandings of disease mechanisms gained in laboratory into the development of new methods for diagnosis, therapy, prevention and their first testing in humans.” The roundtable identified the second roadblock (T2) as “the translation of results from clinical studies into daily clinical practice and health decision making” [6]. Interestingly, this model was highly aligned with the NIH definition. The model portrays T2 as one step—the translation of new knowledge into clinical practice—but the process is rarely that simple**.** Westfall et al. redrew the model including a third step (T3), practice-based research, which is often necessary before distilled knowledge can be implemented in practice [7]. Thus, the second phase of translation was later subdivided to create a model of the translational phases which include basic science to clinical science (T1), to clinical practice (T2), and to health improvements (T3) [8].

D. Dougherty and P. H. Conway proposed the 3 T’s Road Map in 2008 [9]: basic biomedical science to clinical efficacy knowledge (T1), to clinical effectiveness knowledge (T2), and to improved health care quality and value and population health (T3). Next, translation 2 activities focus on creating more patient-specific evidence of clinical effectiveness (T2), as well as comparative effectiveness to identify “the right treatment for the right patient in a right way at the right time” and translation-into-practice guidelines and tools for patients, clinicians, and policy makers. Translation 3 activities comprise the essential third step along the 3 T’s road map (T3). Its activities address the “how” of health care delivery so that evidence-based treatment, prevention, and other interventions are delivered reliably to all patients in all settings of care and thus improve the health of individuals and populations. Meanwhile, the Evaluation Committee of the Association for Clinical Research Training (ACRT) proposed an operational definition to use in the 3 T’s educational framework [10]. They posited that translational research fosters the multidirectional integration of basic research, patient-oriented research, and population-based research, with the long-term aim of improving public health. In this model, T1 research expedites the movement between basic research and patient-oriented research that leads to new or improved scientific understanding or standards of care. T2 research facilitates the movement between patient-oriented research and population-based research that leads to better patient outcomes, the implementation of best practices, and improved health status in communities. And T3 research promotes interaction between laboratory-based research and population-based research to stimulate a robust scientific understanding of human health and disease. This model offers a framework to guide institutions in developing processes of program evaluation.

The most current translation model in the literature expounds the 4 T’s [8]: basic scientific discovery (basic knowledge) to potential clinical application (theoretical knowledge) (T1), to evidence-based guidelines (efficacy knowledge) (T2), to clinical care or intervention (applied knowledge) (T3), and to the health of a community or population (public health knowledge) (T4). In this model, T1 translational research (potential application) is defined as translation of basic research into a potential clinical application. T2 translational research involves efficacy studies in which new therapies are tested under controlled environments to provide the link between potential clinical applications and potential evidence-based guidelines. T3 translational research (effectiveness studies) involves translation from recommendations or guidelines into practice. T4 translational research (population-based) involves outcomes assessments at the community or population level (public health).

Translational medical research has recently experienced an upsurge in interest and funding worldwide. The Director of NIH, Dr. Francis Collins, proposed a new initiative of five thematic areas in 2010, and “Translating Basic Science Discoveries into New and Better Treatments” was one of five thematic areas [11]. Many translational research programs, centers and institutes have been rapidly established and lots of core journals around the world have opened translational medicine columns [3,12], such as *Science Translational medicine research*, the *American Journal of Translational Research*, *Journal of Translational medicine research*, *Translational Research* and *Clinical and Translational Science*. Moreover, Clinical and Translational Science Awards (CTSAs), The Translational Medical Research Award and the Bedside-to-Bench Award were established to encourage translational medical research [13]. By 2012, the CTSA Consortium had expanded to approximately 60 medical research institutions located throughout the nation, linking them together to energize the discipline of clinical and translational science. At the same time, NIH created its newest National Center for Advancing Translational Sciences (NCATS) to advance the development, testing, and implementation of diagnostics and therapeutics across a wide range of human diseases and conditions. Advancing translational sciences has become an important mission of NIH. Translational research has also become a centerpiece of the European Commission for health related research in 2006, and during which, the United Kingdom invested the most to establish translational research centers [14]. The Translational Medicine Research Initiative (TMRI) [15], Scottish Translational Medicine Research Collaboration (TMRC) [16] and the Office for Strategic Co-ordination of Health Research (OSCHR) [17] were established to facilitate more efficient translation of health research into health and economic benefits in the UK. After 2006, many other countries and regions started to establish translational research center for translational research. The People's Republic of China has established more than fifty translational medicine research centers as of 2012.

Despite the fact that translational medicine has developed rapidly worldwide in recently, there have been few attempts to gather data about the worldwide scientific production of translational medical research. Bibliometric research has been recently used as a quantitative analysis method for scientific research evolution in recent years. The derived statistics that measure the contribution of scientific publications within a given topic could represent current research trends and be used to identify focuses of future study [18]. Through a bibliometric research of literature, the next research trend may be predicted [19]. In this study, the records of literature are analyzed using several aspects of bibliometric methodology. The main body of this article includes bibliometric analyses in the publishing year, document type, subject categories, publication distribution, patterns of journals, countries/regions, institutes and authors [20,21]. In addition, appropriate statistical tests are used in the authors’ keyword yearly to predict the developing trend of translational medical research. Moreover, citation data will also be used as a bibliometric tool to indicate the intellectual impact of the research output. We are making efforts to address the following questions regarding the field of translational medical research:

• Growth trend of global publication output from 1993 to 2012.

• Subject categories of publication and the relationship between these subject categories.

• Journals of publication identified.

• Countries of publication and international collaboration.

• Institutes of publication and international collaboration.

• Authorship and co-authorship of papers.

• Citation analysis of research publications.

• Distribution of keywords, MeSH terms, and hot topics.

These efforts will provide evidence of the current status and trends in translational medical research all over the world, as well as clues to the impact of this popular topic, thus helping researchers understand the panorama of global translational medical research, and predict the dynamic directions of research.

### Data and methodology

#### ***Data sources***

As a strictly selected abstract database, Web of Science (WoS, including SCI-E and SSCI) has long been recognized as the most authoritative scientific and technical literature indexing tool providing data on most important areas of science and technology research [22]. We collected the publications on translational medical research using the Web of Science database online version published by Thomson Router ISI, operated by Thomson Scientific, Philadelphia, PA, USA [23]. The main advantage of the ISI journals is that they constitute the most important (in terms of impact) journals in the world [22,24]. Drawing upon relevant research experience [25,26], articles were extracted using text-supplied keywords from the National Library of Medicine’s Medical Subject Headings thesaurus. Terms searched were “Translational Medical Research”, “Translational Medical Research”, “Translational Medicine”, “Translational Medical Science”, “Translational Research”, “Medical Translational Research”, “Knowledge Translation”, etc. Identification of translational medical research articles was accomplished by searching titles, author-supplied abstracts, and texts. The search strategy of WoS was (TS = (“Translat* Medic*” OR “Translat* Research*” OR “Medic* Translat*”) OR SO = (Translat*)) NOT (WC = (Information Science Library Science OR Education Educational Research OR Education Special OR Social Issues OR Social Work OR Computer Science Interdisciplinary Applications OR Social Sciences Interdisciplinary OR Sport Sciences OR Statistics Probability OR Plant Sciences OR Zoology OR Computer Science Information Systems OR History Philosophy of Science OR Ethics OR Computer Science Artificial Intelligence OR Nuclear Science Technology OR Language Linguistics OR Linguistics )). The search strategy of PubMed was “Translational Medical Research”[Mesh] OR “Translational Medical Research*”[Title/Abstract] OR “Translational Medicine”[Title/Abstract] OR “Translational Medical Science*”[Title/Abstract] OR “Translational Research*”[Title/Abstract] OR “Medical Translational Research*”[Title/Abstract] OR “Medical Research, Translational”[Title/Abstract] OR “Research, Translational Medical”[Title/Abstract] OR “Medicine, Translational”[Title/Abstract] OR “Translational Research, Medical”[Title/Abstract] OR “Research, Medical Translational”[Title/Abstract] OR “Medical Science*, Translational”[Title/Abstract] OR“ Research*, Translational”[Title/Abstract] OR “Knowledge Translation*”[Title/Abstract] OR “Translation*, Knowledge”[Title/Abstract].

The benefits of using bibliometric search terms are always debatable. While the identification of the appropriate terms identifying translational medical research may be a matter for further studies, we suggest that these terms provide an adequate balance for the objectives of this investigation. They are in accordance with previous investigations as discussed in the methodology section. A total of 5500 publications were identified in the SCI and SSCI databases and 5,452 publications were identified in PubMed database within all timespans. The impact factor (IF) of WoS journals in 2012 was determined by Journal Citation Reports (JCR), which was the latest data available. Papers originating from England, Scotland, Northern Ireland, and Wales are grouped under the UK heading, while those from Hong Kong, Macao, and Taiwan are not included under the China heading.

## Methods

One of the earliest definitions of bibliometric describes it as “the application of statistical and mathematical methods to books and other media of communication” [27]. Today, bibliometric is often used to assess scientific research through quantitative studies on research publications. Bibliometric assessments are based on the assumption that most scientific discoveries and research results are eventually published in international scientific journals where they can be read and cited by other researchers [28]. In this paper, the distribution of the publishing year, document types, language, subjects, journals, countries, institutions, times cited frequency of keywords, cluster analysis as well as collaboration of the WoS papers were thoroughly examined. The Thomson Data Analyzer (TDA), VOSviewer and Aureka software were employed to analyze the publications for knowledge mapping. The Thomson Data Analyzer™ desktop software often offers a powerful function for managing and extracting scientific data within databases. It gives a statistical results for research work [29]. VOSviewer is a computer program that can create maps based on network data. It can view and explore maps written in the Java programming language and can operate on most hardware and operating system platforms. VOSviewer is primarily intended to be used for analyzing bibliometric networks [30]. The program can be used to create maps of publications, authors, or journals based on a co-citation network or to create maps of keywords based on a co-occurrence network. And with Aureka, one can study the full text of millions of global literature, maximize the top-line revenue its portfolio generates, and visualize data to reveal trends and opportunities [31,32].

The term “co-author,” used to denote multiple writers appearing simultaneously in one paper, also reflects the collaboration of different institutes, regions, or countries [33,34]. The higher the strength of these co-authorships, the closer the relationship among them is. Collaboration between countries was determined by the author description, where the term “independent” was assigned if no collaboration was presented. “International collaboration” was assigned to research if it was co-signed with researchers from more than one country. “Co-words” refers to the phenomenon that two or more keywords occur simultaneously in one article or one field, where the number of times cited is called the frequency or strength of co-words [35]. “Cluster analysis” is a collective term covering a wide variety of techniques for delineating natural groups or clusters in data sets [36]. The task of it is to group a set of objects in such a way that objects in the same group (called clusters) are more similar to each other than to those in other cluster groups. It was used in many fields, including machine learning, pattern recognition, image analysis, information retrieval, and bioinformatics [37,38]. In this study, co-author, co-word, and cluster analysis methods were used to analyze the collaboration among several research organizations through visualization technology, which was also called knowledge mapping technology [39,40]. Knowledge mapping contain scientific data gathering, surveying, exploring, discovery, conversation, disagreement, gap analysis, education and synthesis technologies. It aims to track the loss and acquisition of information & knowledge, personal and group competencies and proficiencies, show knowledge flows, appreciate the influence on intellectual capital due to staff loss, assist with team selection and technology matching. The map of the keywords can forecast the future trend of a science subject well [41].

## Results and discussion

Figures and tables were employed to describe the production and future trends of translational medical research. Papers from the WoS database were studied carefully using bibliometric analysis.

There are 5,500 total translational medical research-related papers in the WoS database, including 16 document types. Following the conventions used in other bibliometric studies, we restrict further analysis to articles, which are peer-reviewed and represent original scientific development. Publications of all other types are thus removed from the analysis for the rest of this article. As for the publishing language, 3,197 or 97.9% of the 3,267 journal articles are written in English. These figures confirm that English is the prevalent academic language, and most SCI and SSCI indexed journals are published in English.

### Global publication trend

**Figure 1:**
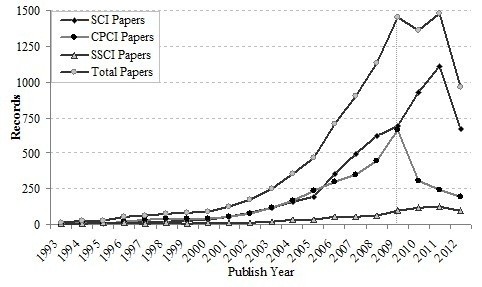
Research papers published from 1993 to 2012.

The trend of annual papers in translational medical research from 1993 to 2012 is shown in Figure 1. During the past two decades WoS (including SCI, SSCI and CPCI) papers on translational medical research produced ranged from five in 1993 to approximately 1,500 in 2011. WoS annual number of publications has grown exponentially, especially after 2001, indicating that the research has recently garnered more attention. It can be seen from Figure 1. that not many researchers pay attention to the translational medical research before 2001, and only a few papers were published. After 2006, the number of SCI papers rapidly increased and reached its production peak in 2011. The number of CPCI papers started to decrease from 2009, while the number of SSCI papers increased slowly during the last two decades.

### Subjects of research papers

**Figure 2:**
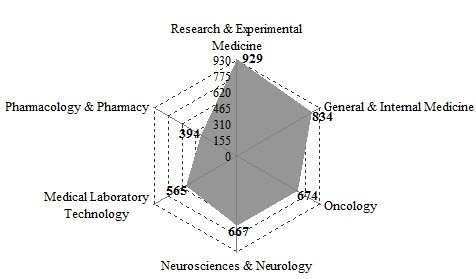
Main subjects of WoS papers on translational medical research.

The top six subject categories based on the Journal Citation Report (JCR) are shown in Figure 2. They are all branches of medical science. Figure 2. also shows that translational medical research was mainly located in fields of basic clinical science (including Research & Experimental Medicine, Medical Laboratory Technology) and clinical medical science (including General & Internal Medicine, Oncology, Neurosciences & Neurology), where Pharmacology & Pharmacy also play an important role. In addition, more studies have focused on public health science, including Public, Environmental & Occupational Health and Health Care Sciences & Services, wherein investigators study factors and interventions that influence the health of populations to improve public and global health. Figure 2. shows that public health research had become an important part of translational medical research.

**Figure 3:**
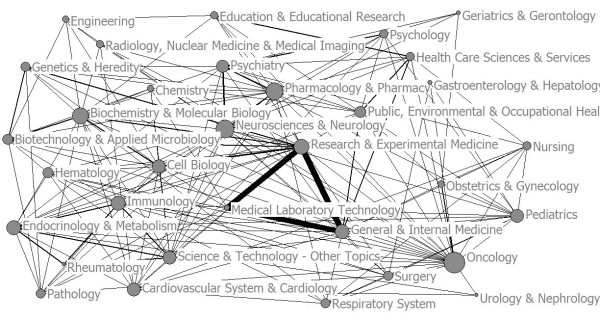
Subjects categories co-occurrence network.

To represent more synthetically the relations between categories, the subjects’ categories co-occurrence network was drawn and visualized in Figure 3. In this network map, the centrality of a node representing a subject category is a graph-theoretical property that quantifies the importance of the node’s position in a network. Figure 3 shows that in the scientific network map of translational medical research, the centrality of the terms “Research & Experimental Medicine”, “Medical Laboratory Technology”, “General & Internal Medicine” is outstanding. Meanwhile, the position of “Oncology”, “Neurosciences & Neurology”, “Pharmacology & Pharmacy”, “Cell Biology” “Biochemistry & Molecular Biology”, “Immunology” are very important. These subjects play a key role in translational medical research.

### Journals of publication

The top 11 journals with more than 25 articles are displayed in Table 1. Approximately 23% of WoS papers resided in these most productive top 11 journals, which are considered the core journals of translational medical research area under Bradford Law [42]. The remaining journals make up a smaller percentage.

**Table 1:** Basic information of top productive journals

**No**	**Journal**	**Records**	**Country**	**IF**^1^**(2012)**
1	Translational Research	323	USA	3.490
2	Science Translational Medicine	86	USA	10.757
3	CTS-Clinical and Translational Science	65	USA	2.330
4	Journal of Translational Medicine	48	England	3.459
5	Journal of Investigative Medicine	37	USA	1.746
6	Clinical Cancer Research	35	USA	7.837
7	Diabetes Care	34	USA	7.735
8	Academic Medicine	34	USA	3.292
9	European Journal of Cancer	33	England	5.061
10	PLoS One	27	USA	3.730
10	Annals of Oncology	27	England	7.384

^1^ IF is impact factor, devised by Eugene Garfield, is calculated yearly starting from 1975 for those journals that are indexed in the Journal Citation Reports. The impact factor (IF) of an academic journal is a measure reflecting the average number of citations for recent articles published in the journal. It is frequently used as a proxy for the relative importance of a journal within its field, journals with higher impact factors deemed to be more important than those with lower ones.

**Figure 4:**
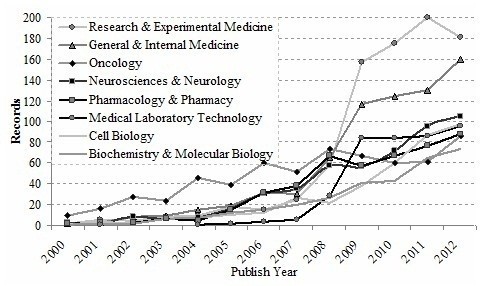
Annual journals distribution of WoS papers.

The annual distribution of these papers is noted in Figure 4. It can be seen that the *Translational Research* and *Science Translational Medicine* published numerous articles from 2007 to 2011. *Translational Research*, one of journals supported by the CTSA, was renamed from the *Journal of Laboratory and Clinical Medicine* in 2006. *Science Translational Medicine* had a drastic increase in article publication during this period. However, publication of other journals increased slowly while its growth rate remained. According to these trends it may be possible that *Translational Research*, *CTS,* and *Science Translational Medicine* will be the primary journals for translational medical research publication in coming years.

### Countries of publication

Top countries of publication of these papers are noted in Table 2. There were 74 countries/territories producing WoS papers on translational medical research over the investigation period. Among them, only nine countries had article output during the 1992–2001 timeframe, and 65 countries/territories had just began to publish papers after 2001. The top 10 countries/territories were ranked by number of articles (Table 2). Two North and Central American countries, six European countries, and two Asian countries/territories were ranked in the top 10 for WoS papers published. There are no African countries in the top 10. The seven major industrialized nations of the world (G7 countries [2] ), the USA, UK, Germany, Canada, Italy Japan, and France, were the top seven countries for publication. The pattern of domination in publication of the G7 countries has occurred in most scientific fields [43]. It reflects the high economic activity and academic level of these countries [44]. The USA had the greatest contribution with 2,752 papers. WoS publications from UK, Germany, Canada, Italy, and Japan are 466, 333, 307 266, and 205 respectively. The number of publications from other countries was below 200. Japan was the sixth most-active country according to the total papers published. The top two productive countries carried out most of the international collaborations with others in the translational medical research field (see Table 2).

**Table 2:** Publication distribution in top countries

**Country**^**2**^	**USA**	**UK**	**Germany**	**Canada**	**Italy**	**Japan**	**France**	**P.R China**	**Netherlands**	**Spain**
**Records**	2752	466	333	307	266	205	160	158	157	152
**Percentage**	53.74	9.09	6.50	6.00	5.20	4.00	3.12	3.09	3.06	2.97

^2^ The G7, as a group consisting of the finance ministers of seven industrialized nations: the U.S., U.K., France, Germany, Italy, Canada and Japan, is seven of the eight (China excluded) wealthiest nations on Earth, not by GDP but also by global net wealth.

**Figure 5:**
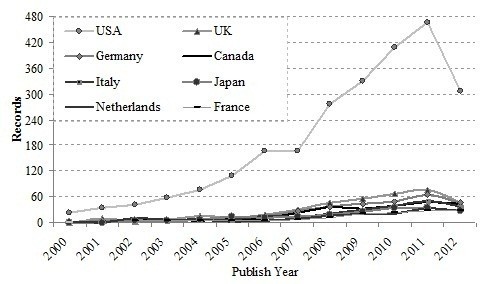
Annual WoS publications distribution of top 10 countries.

Figure 5 shows the published papers of the most productive countries during the period of this study. It can be concluded that translational medical research started in most countries in the 1990s and increased after 2005. The USA began its translational medical research earlier and kept rapidly increasing paper production. Compared with UK, Germany started earlier but dropped behind thereafter. The USA was the leading country during last decade, where papers published from 2000–2011 increased dramatically from 11 to 518 during the last decade. It can be said that translational medical research is still a hot field in today’s world and is likely to continue in popularity.

**Figure 6:**
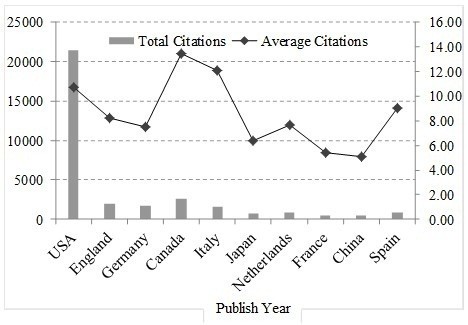
Citations of WoS papers for top 10 countries.

The top countries’ paper citations are noted in Figure 6. It shows information about the total citation and citation frequencies of the research papers from the top countries in the global fields of translational medical research. It can be seen that the total citation count of the USA is highest, followed by UK, Germany, Canada and Italy in turn. The average citation frequency, sorted in descending order, is Canada, Italy and the USA. The USA and UK are on the front ranks except for average citation frequency, showing their superiority in translational medical research. Canada ranks fourth in issued number of papers and first in article citation frequency, which indicates the high average quality of the papers. Japan and China rank sixth and ninth respectively in issued volumes with lower article citation frequencies, which may indicate that there is a considerable problem with the quality of Japanese and Chinese papers issued.

### Institutes of publication

**Figure 7:**
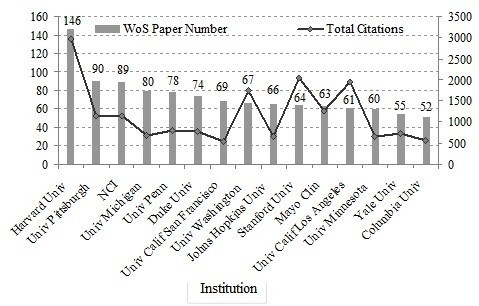
The institutes distribution of WoS papers.

The contribution of different institutes was assessed by the institute of the affiliation with at least one author in the published papers. The top 15 institutes with paper quantity of more than 50 were ranked by their published papers. According to Figure 7, Harvard University and the University of Pittsburgh performed well: they are two of the most powerful institutions in translational medicine research. Harvard University published 146 papers, ranking first and followed by the University of Pittsburgh with 90 papers. It can be seen that the total citation count from Harvard University is the highest, followed by Stanford University and the University of California Los Angeles. Harvard University was the leading institute both in paper quantity and quality. All the organizations belong to the United States; reflecting the overall strength of the U.S. universities.

### Citation of research papers

The number of citations does not necessarily indicate the quality of a paper, but it is a measure of its impact and/or visibility in translational medical research field. The top 10 most frequently cited articles (≥40 times) from 1992 to 2012 were selected in Table 3. The most frequently cited article was “The Meaning of Translational Research and why it Matters,” published in 2008 by S. H. Woolf. It has been cited 111 times since it was published in the *JAMA*, which vastly exceeds the citation of the others articles on the translational medical research field. E. A. Zerhouni, at the National Institutes of Health contributed the highest number (No. 3 and 4) of articles among the 10 most frequently cited articles, which exhibits its predominance. These top cited papers were all authored by scientists from institutions in developed countries. The USA contributed ten and the Netherlands contributed one.

**Table 3:** **Top 10 most cited WoSpapers**

**No.**	**Time cited**	**Authors**	**Title**	**Journal**	**Institute**	**Country**	**Year**
1	111	Woolf Steven H.	The meaning of translational research and why it matters	JAMA	Virginia Commonwealth University	USA	2008
2	102	Sung, NS; Crowley, WF; Genel, M; et al.	Central challenges facing the national clinical research enterprise	JAMA	Burroughs Wellcome Fund	USA	2003
3	66	Zerhouni, EA	Translational and clinical science - Time for a new vision	New EnglandJournal of Medicine	National Institutes of Health	USA	2005
4	65	Zerhouni, EA	The NIH roadmap	Science	National Institutes of Health	USA	2003
5	60	Westfall, JM; Mold, J; Fagnan, L	Practice-based research - “Blue Highways” on the NIH roadmap	JAMA	University of Colorado Health Sciences Center	USA	2007
6	53	Hanahan, D; Weinberg, RA	The hallmarks of cancer	Cell	University of California, San Francisco	USA	2000
7	46	van de Vijver, MJ; He, YD; van ’t Veer, LJ; et al.	A gene-expression signature as a predictor of survival in breast cancer.	New EnglandJournal of Medicine	Netherlands Cancer Institute	Netherlands	2002
8	44	Marincola FM	Translational medical research: A two-way road	Journal of Translational medical research	National Institutes of Health	USA	2003
9	41	Khoury, MJ; Gwinn, M; Yoon, PW; et al.	The continuum of translation research in genomic medicine: how can we accelerate the appropriate integration of human genome discoveries into health care and disease prevention?	Genetics in Medicine	National Office of Public Health Genomics Centers for Disease Control and Prevention	USA	2007
10	40	van’t Veer, LJ; Dai, HY; van de Vijver, MJ; et al.	Gene expression profiling predicts clinical outcome of breast cancer	Nature	Rosetta Inpharmat, Kirkland	USA	2002
10	40	Paez, JG; Janne, PA; Lee, JC; et al.	EGFR mutations in lung cancer: Correlation with clinical response to gefitinib therapy	Science	Departments of Medical Oncology and Cancer Biology, Dana-Farber Cancer Institute	USA	2004

Table 3 also shows that most articles focused on the meaning and importance of translational medical research, and highlights the “NIH roadmap” and CTSAs. The NIH Roadmap is a set of bold initiatives aimed at accelerating medical research that addresses challenges that no single NIH institute could tackle alone, but the agency as a whole must undertake. The Roadmap identifies the most compelling opportunities in three arenas: new pathways to discovery, research teams of the future, and reengineering the clinical research enterprise. As early as 2006, the NIH made translational research a priority, forming centers of translational research at its institutes and launching the Clinical and Translational Science Award (CTSA) program. By 2012, the NIH had founded 60 research centers with a budget of $500 million per year. Besides academic centers, foundations, industry, disease-related organizations, individual hospitals and health systems have also established translational research programs, and at least two journals (*Translational Medicine* and the *Journal of Translational Mdicine*) are devoted to the topic. Other most frequently cited articles prove that researchers from around the world have concentrated on translation research of biomarkers, genomics gene-expression for health care and disease prevention. From the data it can be concluded that the translational medical research trend will focus on basic medicine, clinical medicine, and public health.

### Authorship and co-authorship

**Figure 8:**
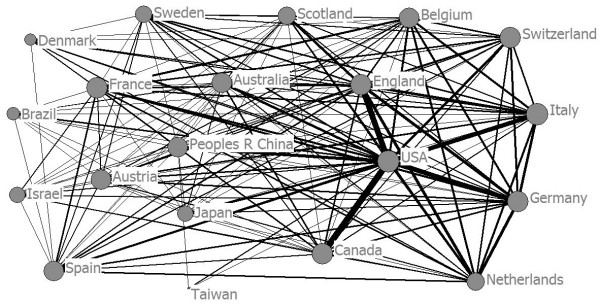
Co-authorship for WoS publications in different countries.

Owing to the specialization of research activities of each nation, international collaboration could be effective in promoting the creation, transmission, and sharing of knowledge, and in posing a serious obstacle to the diverse types of collective, exchangeable, and integrated knowledge [45]. Increasing globalization may lead to the increase of international collaboration in science and technology. The national cooperative network map of translational medical research has been drawn according to the national/regional cooperation data. As shown in Figure 8, a total network of higher density illustrates closer cooperation between countries. The USA, UK and Canada cooperate frequently with other countries/regions and stand at the core position of the entire network, which in turn benefits from their knowledge transfer among translational medical researchers. The USA, which has issued the most volumes, and other nations such as Australia, PR China, and Japan, France, Austria Switzerland, Italy, Germany, Belgium, and Netherlands, are in the peripheral layer. Other countries/regions such as Taiwan and Brazil have had less cooperation with other regions/countries, so they are in the outermost layer of the entire cooperation network.

**Figure 9:**
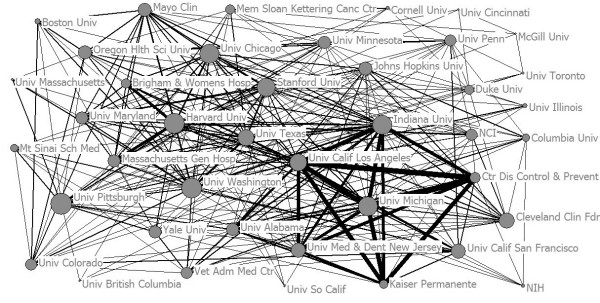
Co-authorship for WoS publications in different institutes.

Collaboration within one organization is found to be as high as 41.49%. Collaboration with authors from more than one institute is in the majority, which covers more than one half of the figures. From this data, it can be concluded that collaboration between institutes is in the mainstream of translational medical research. The cooperative network map of organizations in translational medical research is also drawn according to the cooperation between institutions. The American institutes of Harvard University, the University of Michigan, the University of California Los Angeles, and Indiana University are in the core status of the network; these institutions cooperate with other organizations frequently, thus playing an important role in the process of knowledge transfer among organizations. Other high-yield institutions such as Canada’s University of Toronto, McGill University, and the University of British Columbia are at the edge of the network due to their less cooperation. It can be concluded that American institutions have made great advances in paper production and cooperation, with great strength and good development prospects. The annual count of authored papers is noted in Figure 9.

**Figure 10:**
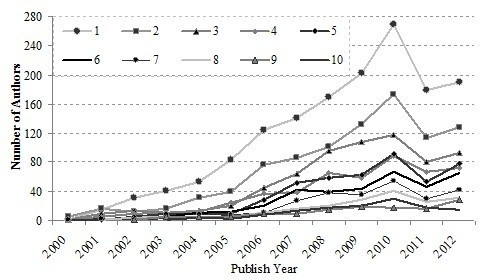
Annual number of authors of WoS papers.

As shown in Figure 10, most work on the translational medical research was done by one to five researchers, most notably by one scientist, a mainstream trend of all research activity. Some of the translational medical research studies were the results of collaboration by more than five authors. This level of collaboration between scientists or their institutes is indeed the trend of modern research activity. Most translational medical research was done by the collaboration of several scientists. It can be seen from Figure 10. That the one to three scientists’ teams increased rapidly after 2005, while five-person or four-person teams increased quickly, but were not as steady compared to others. Convenient modern communications make these collaborations possible. Single author articles increased from 2000 to 2005 in linear expansion, but didn’t increase as fast as papers published by a few co-authors after 2005. The analysis indicates that collaboration is not only possible but necessary for the translational medical research.

### Keywords and co-words

**Figure 11:**
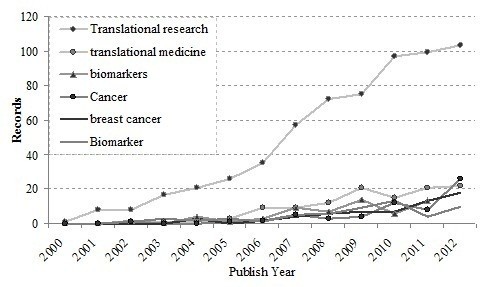
Timeframes of keywords in the period of 2001–2011.

The technique of statistical analysis of keywords and title-words may indicate directions of research. Especially, authors’ keywords analysis could offer information on research trends as viewed by researchers [19]. The high percentage of once-only author keywords probably indicates a lack of continuity in research and a wide disparity in research focuses. Another reason is that these keywords might not be standard or widely accepted by researchers. Author keywords appearing in the articles referring to translational medical research from 1993 to 2012 were calculated and ranked. The top eight author keywords used and distribution during the last 20 years are displayed in Figure 11, where research variations can be roughly grasped. Except for “translational research,” and “translational medicine” which were search words, the top six most frequently used keywords were “biomarkers”, “stroke”, “inflammation”, “cancer”, “biomarker”, and “breast cancer.”

Figure 11 shows the most frequently used author keywords distributed from 2000 to 2012. During the past decade the keyword “translational research” rose from only several times mentioned to more than 100 times, there by becoming the most dominant keyword used in the study. Other than “translational medicine,” “biomarkers”, “inflammation”, “cancer”, “biomarker”, and “breast cancer.” had high increasing rates. These figures reveal that “biomarker/ biomarkers” are hot topics in translational medical research throughout the world, and are of serious concern to researchers. Biomarker and cancer research may thus play important roles in translational medical research science in the future. Otherwise, researchers are making progress across a broad range of diseases and conditions, such as cancer (especially breast cancer), diabetes, neurological disorders, and heart disease, which are the main application fields of translational medical research.

**Figure 12:**
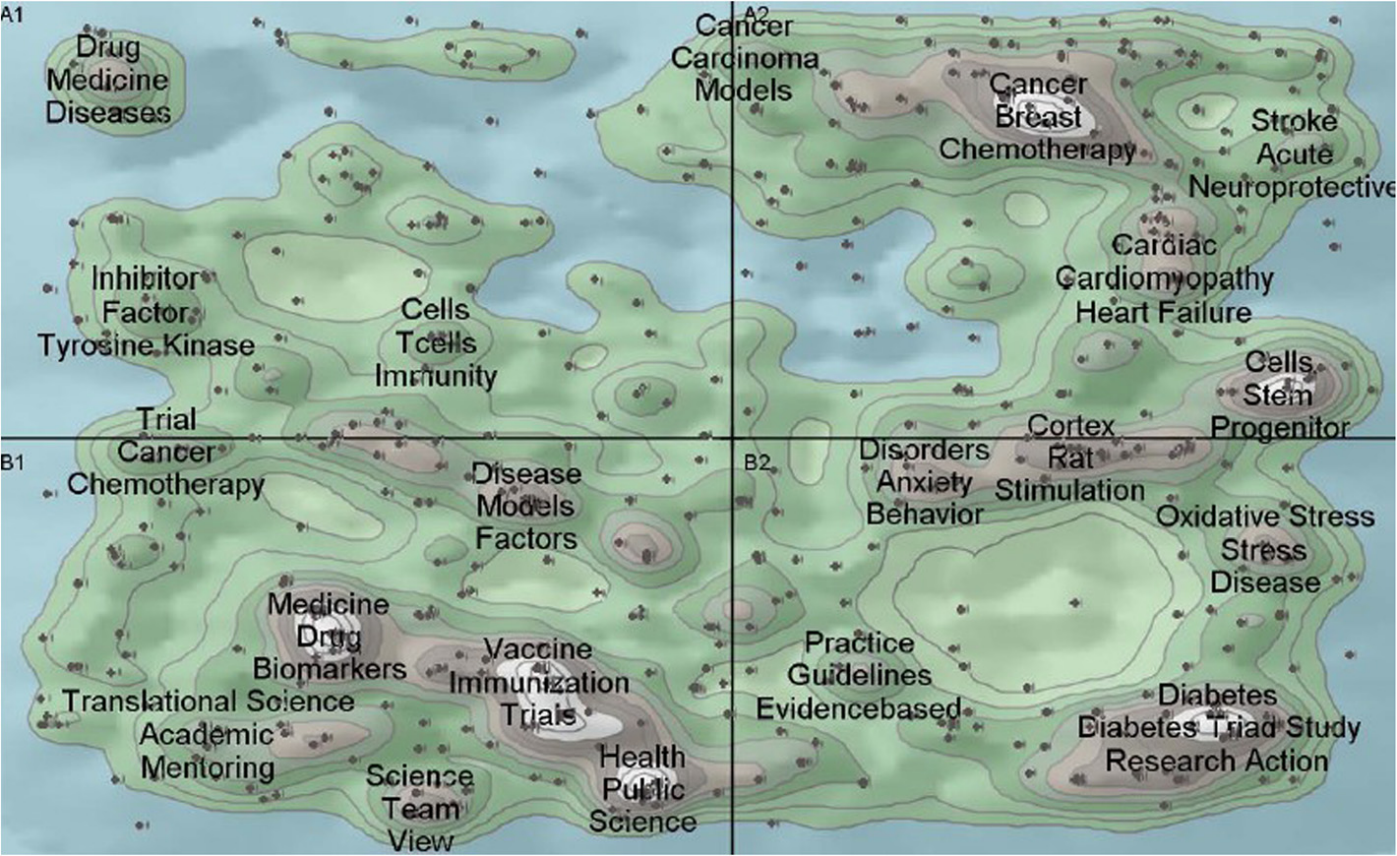
Clusters and Co-words Map of WoS papers.

The title of an article has the core information that authors would like to express [46]. Meanwhile, author keyword analysis offers information about research trends from the view of researchers, and has proved to be important for monitoring the development of science [47]. Keywords Plus supplied additional search terms extracted from the titles of articles cited by authors in their bibliographies and footnotes [48]. The topic of papers can be obtained from the title-words, author keywords, and keywords plus by cluster analysis. Clusters or co-words maps of papers could image the core competency of the translational medical research. Figure 12 is derived from bibliometric analysis on the papers of translational medical research in WoS by Aureka software. It can be seen that these topics listed below are topics in translational medical research: drug design and development, pathogenesis and treatment of disease (such as cancer, acute stroke, cardiac disease, disorders, stress disease, and diabetes), disease model research, evidence-based research (such as guideline practice and nurse practice), stem and progenitor cells, immunity and vaccine, biomarkers, training and career development of scientists, fostering collaborations and research teams, and public health science.

**Figure 13:**
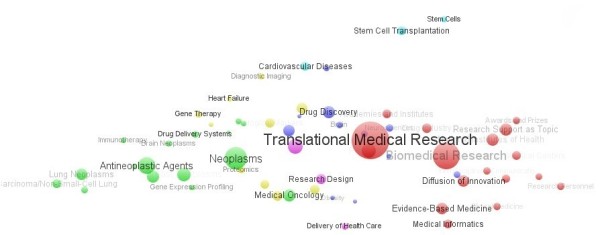
Clusters and Co-words Map of PubMed papers.

Figure 13 shows the clusters of the top 60 most-used MeSH terms in the 20 years. The most frequently used keyword was “Translational Medical Research” as it was the string used for searching in this study. Similarly with the cluster results of title-words, author keywords and keywords plus from WoS, “drug design/drug discovery”, “antineoplastic agents”; “neoplasms”, “medical oncology”, “lung neoplasms, carcinoma/non-small-cell lung”, “brain neoplasms”, “prostatic neoplasms”, “tumor markers/biological”; “biological markers”; “immunotherapy”, “heart failure”, “cardiovascular diseases”; “stem cell transplantation, stem cells”, and “evidence-based medicine, medical informatics” also appeared in the clusters of MeSH Major Topic (Figure 13). Besides, the MeSH Major Topic related to neoplasms diseases such as “proteomics”, “gene expression profiling”, “gene therapy”, and “research design” were given more attention during the last 20 years, which showed that they may be new methods used to treat cancer and other diseases. Furthermore, “diagnostic imaging”, “obesity”, “delivery of health care”, “biomedical research, diffusion of innovation, interdisciplinary communication” and “National Institutes of Health (U.S),research support as topic, awards and prizes”, which did not appear in Figure 13, also showed a hot topics in translational medical research fields.

## Conclusion

This study summarized some significant research trends and performance in worldwide translational medicine.The articles on translational medicine increased rapidly during the last 20 years. Translational medical research articles were mainly located in the fields of Research & Experimental Medicine, General & Internal Medicine, Oncology, Neurosciences & Neurology, Medical Laboratory Technology, and Pharmacology & Pharmacy. Meanwhile, “Research & Experimental Medicine”, “Medical Laboratory Technology”, and “General & Internal Medicine” play a key role in translational medical research subjects categories co-occurrence network. Moreover, more attention was paid to Public, Environmental & Occupational health and Health Care Sciences & Services in recent years. It is clear that translational medicine will be a focus in the public health field besides basic clinical science and clinical medical science in future. The top three most productive journals, which are *Translational Research (323), Science Translational Medicine (86) and CTS (65)*, published approximately 14.5% of WoS papers. Especially *Translational Research*, renamed from the Journal of Laboratory and Clinical Medicine, was the chief journal for the translational medicine research in last three years. Meanwhile, *Science Translational Medicine*, as sub journal of *Science*, has the highest impact factor among translational medical research journals. The G7 countries, the USA, UK, Germany, Canada, Italy, Japan, and France, are the leaders of translational medical research. The USA and the institutions of America play a dominant role in the production, collaboration, citation and high quality articles. In the perspective of collaboration, the research papers were mainly completed by one to five authors, and Multi-authors comprised a larger percentage.

Except for “translational research” and “translational medicine”, the terms most frequently used in the last decades of research were “biomarker”, “stroke”, “cancer” and “breast cancer”. This suggests that “biomarker” and “genomics” in disease research and application (such as cancer, diabetes, etc.) are the mainstream topics in the study field. Analyzing the keywords distribution trends shown, it can be assumed that translational research will attract more research interest. Recent major topics of translational medical research included drug design and development, pathogenesis and treatment of disease, disease model research, evidence-based research, stem & progenitor cells, immunity & vaccine, biomarkers, training and career development of scientists, fostering collaborations. Besides, the translation among clinical medical science, basic clinical science, and public and health science may be new research direction, especially the T3 (Efficacy. Potential clinical application (theoretical knowledge) to evidence-based guidelines (efficacy knowledge)) and T4 (Clinical care or intervention (applied knowledge) to the health of a community or population (public health knowledge)) researches. The findings of this study can help scientific researchers understand the performance and central trends of translational medical research globally and therefore suggest directions for further research.

### Limitations

Relevant articles were extracted using text-supplied keywords from the National Library of Medicine’s Medical Subject Headings thesaurus. In order to improve the two criteria, papers in journals whose name contains “translat*” are also included, and the papers which do not belong to medicine-related subjects are excluded. However, the benefits of using bibliometric search terms are debatable. The identification of the appropriate terms identifying translational medical research may be a matter for further studies. Due to limited resources and research levels, this study only searched sound articles in the Web of Knowledge and PubMed, which content has certain limitations. Moreover, methods of social network analysis and visualization technologies are relatively fresh perspective but lacking innovation for its newly application in this field. In addition, the lacking of regularity in the key word (extracting 20% keywords according to the law of two to eight) and mesh term selection also impacts the analysis process and results.

## Competing interests

The authors declare that they have no competing interests.

## Authors’ contributions

QY and PHL created this study. QY, PHL and LY were involved in the data analysis. QY, PHL, FCM and SJZ contributed to the research design. SJZ obtained funding. All authors were involved in the interpretation of data and have read and given final approval of this paper.

## Pre-publication history

The pre-publication history for this paper can be accessed here:

http://www.biomedcentral.com/1472-6947/13/77/prepub
